# Impact Properties and Water Uptake Behavior of Old Newspaper Recycled Fibers-Reinforced Polypropylene Composites

**DOI:** 10.3390/ma13051079

**Published:** 2020-02-28

**Authors:** David Hernández-Díaz, Ricardo Villar-Ribera, Francesc X. Espinach, Fernando Julián, Vicente Hernández-Abad, Marc Delgado-Aguilar

**Affiliations:** 1 Serra Húnter Programme, Department of Graphical Engineering and Design, Universitat Politècnica de Catalunya, Campus Terrassa, 08222 Terrassa, Spain; david.hernandez-diaz@upc.edu; 2 Department of Graphical Engineering and Design, Universitat Politècnica de Catalunya, Campus Manresa, 08242 Manresa, Spain; villar@ege.upc.edu; 3 Design, Development and Product Innovation, Department of Organization, Business, University of Girona, 17003 Girona, Spain; fernando.julian@udg.edu; 4 Department of Graphical Engineering and Design, Universitat Politècnica de Catalunya, Campus Terrassa, 08222 Terrassa, Spain; vhdez@ege.upc.edu; 5 LEPAMAP Group, Department of Chemical Engineering, University of Girona, 17003 Girona, Spain; m.delgado@udg.edu

**Keywords:** lignocellulosic fibers, polypropylene, green composites, recycling, water uptake, impact properties

## Abstract

Natural fiber-reinforced thermoplastic composites can be an alternative to mineral fiber-based composites, especially when economic and environment concerns are included under the material selection criteria. In recent years, the literature has shown how lignocellulosic fiber-reinforced composites can be used for a variety of applications. Nonetheless, the impact strength and the water uptake behavior of such materials have been seen as drawbacks. In this work, the impact strength and the water uptake of composites made of polypropylene reinforced with fibers from recycled newspaper have been researched. The results show how the impact strength decreases with the percentage of reinforcement in a similar manner to that of glass fiber-reinforced polypropylene composites as a result of adding a fragile phase to the material. It was found that the water uptake increased with the increasing percentages of lignocellulosic fibers due to the hydrophilic nature of such reinforcements. The diffusion behavior was found to be Fickian. A maleic anhydride was added as a coupling agent in order to increase the strength of the interface between the matrix and the reinforcements. It was found that the presence of such a coupling agent increased the impact strength of the composites and decreased the water uptake. Impact strengths of 21.3 kJ/m^3^ were obtained for a coupled composite with 30 wt % reinforcement contents, which is a value higher than that obtained for glass fiber-based materials. The obtained composites reinforced with recycled fibers showed competitive impact strength and water uptake behaviors in comparison with materials reinforced with raw lignocellulosic fibers. The article increases the knowledge on newspaper fiber-reinforced polyolefin composite properties, showing the competitiveness of waste-based materials.

## 1. Introduction

The interest toward composites made from thermoplastic matrices reinforced with natural fibers such as flax, hemp, jute, kenaf, and sisal has increased considerably during the recent years. Its use as an alternative to mineral fiber-reinforced materials has increased in the automotive and construction industries. This increase has been boosted by new environmental regulations that promote recycling or the emergence of green certifications and ecolabels. Nonetheless, economic factors must be also taken into account [1]. Precisely, the availability of waste natural fibers from agroforestry or textile, with low or null values, opens the opportunity to create value and expand the value chain of the agroforestry and textile industries [2].

Natural fiber-reinforced composites must be able to replace commodities such as glass fiber-based materials in order to ensure their adoption by the industry [3]. While glass fiber is widely used by the industry, it is more expensive and more difficult to recycle than natural fibers [4]. Thus, under environmental criteria, natural fibers have been postulated as a rational replacement for mineral fibers. Moreover, glass fibers have been proved to be unhealthy, as they can cause skin irritation and even cancer if inhaled [5]. The literature points out the impact strength of natural fiber-reinforced composites as one of the main barriers to their industrial use and notes that upgrading such impact strength will grant their industrial adoption [1,6,7]. On the other hand, the use of hydrophilic reinforcements [8] adds uncertainty regarding the durability of the composites under humid atmospheres, such as outdoors [2]. Not in vain, the mechanical properties of natural fiber-reinforced composites are heavily affected by water absorption [4,9]. Therefore, the impact strength and water uptake behavior of natural fiber-reinforced composites must be researched in order to ensure the competitiveness of such materials.

The impact strength of short fiber-reinforced composites is related to the energy devoted to the break and separation of both phases, and it is also due to fiber slippage [10]. This fiber slippage mechanism has been identified as highly energy consuming and it is the main responsible for increasing the impact strength of the composites [11]. In the case of notched specimens, the impact strength has been also related with the energy devoted to flexural deformations [12].

There are some aspects that affect the impact strength of short fiber-reinforced composites [13]. The strength of the interface between the matrix and the reinforcement is one of the most cited in the literature [11]. The most relevant factors that reduce the impact strength of natural fiber-based composites are the presence of fiber agglomerates that hinder a proper matrix wetting [14,15,16], the stiffness of the matrix [16], the increased stress at the fiber’s end [15], and the hydrophobicity of the matrix in front of the hydrophilicity of the reinforcements [17]. Additionally, the intrinsic mechanical properties of the phases, the morphology of the reinforcements its dispersion, and the mean orientation are also relevant [11].

The use of coupling agents has proved helpful in order to increase the tensile strength of composite materials as well as their impact strength and durability. Coupling agents promote the chemical interactions between fiber surfaces and the matrix by creating hydrogen bonds [18,19]. These chemical interactions increase the strength of the interface and affect noticeably and positively the impact strength and the load transfer between the phases [15]. In this sense, the un-notched impact strength of coupled composite materials was noticeably higher than uncoupled ones [7]. Moreover, the presence of coupling agents improves fiber dispersion [20], preventing the creation of fiber bundles, and thus increasing the impact strength of the materials. Nonetheless, the percentage of coupling agent must be correctly tuned, as excessive amounts of such reactive tend to decrease the impact strength of the materials [10,21]. This can be due to the self-bonding between the chains of the coupling agent, decreasing the interactions with the fiber surfaces and thus affecting the strength of the interface. Therefore, the amount of coupling agent must be studied to optimize the strength of the interface.

The impact strength of short fiber-reinforced composites has been of interest mainly due to the effect of the reinforcements on such property. The literature shows how increasing the reinforcement contents can deliver increases or decreases in the impact strength of a composite material [7]. Furthermore, the relation between the percentage of reinforcement and the impact strength is not linear, and the strength can increase up to a certain reinforcement percentage and decrease when the reinforcement content is further increased [10,11]. The authors relate this behavior with pullout phenomena and energy dissipation at the interface region. A great majority of the authors stressed the importance of the strength of the interface on the impact properties of the materials [22,23,24,25]. The effect of the strength of the interface on the impact strength of natural fiber-reinforced composites has been largely studied. Kenaf fiber-reinforced Poly(Lactic Acid) composites showed an increase of their tensile strength when bleached fibers were used instead of unbleached ones [26]. Bleaching eliminates lignin from the fiber surface, increasing the percentage of celluloses and holocellulose [27], increasing the chemical interactions between the fiber and the matrix, and thus increasing the strength of the interface. This increased interface strength had a noticeable effect on the impact strength of the composites [26]. The aspect ratio of the reinforcements (ratio between the mean length and diameter) has a noticeable effect of the tensile strength of the composites. In this sense, the literature shows how composites reinforced with rice husk powder tend to decrease the tensile strength of the matrix abruptly [28]. In the same paper, an enhanced interface rendered less abrupt decreases. To the best of the authors’ knowledge, the impact strength of natural fiber-reinforced PP composites has been researched, obtaining notable results [7,11]. Nonetheless, the information on the impact strength of newspaper fiber-reinforced composites is scarce [29,30]. Thus, adding further results that stress the capabilities of a source of recycled fibers from waste has importance.

The water uptake of natural fiber-reinforced composites has been profusely studied, and the models that govern the water absorption and diffusion are well established [31,32,33]. The literature is interested in the identification of the diffusion behavior, of which Fickian diffusion is the most common [22,24,34]. The literature is also devoted to the impact of water uptake on the mechanical properties of the composites, as it is one of the main drawbacks of natural fiber-reinforced composites [35,36,37]. Similar to the impact strength, the literature on the water uptake of old newspaper-reinforced polyolefin is scarce [36]. One interesting paper explored the hydrophobic modifications of old newspaper fibers, obtaining reductions in the water absorption rates of the materials [30]. In the present paper, the authors try to avoid using reagents, which is in agreement with the principles of green chemistry [38].

In this work, composite materials prepared with a polypropylene matrix reinforced with different percentages of fibers from old newspapers were formulated and mixed. Two batches for every formulation were prepared: one adding coupling agents and the other uncoupled. Standard impact test specimens were mold injected. The specimens were impact tested. Un-notched specimens were prepared to study the effect of the energy needed to create the fracture on the impact strength of the composites. Besides, the water uptake behavior of the materials was studied. The impact strength results were compared with those of glass fiber-reinforced polypropylene composites. The water absorption was compared with that of the matrix. Previous studies reported some mechanical properties of old newspaper fiber-reinforced polypropylene composites, assessing their competitiveness in front of other materials like other natural fibers and glass fiber (GF) [39,40,41,42,43], but to the best of the authors’ knowledge, the impact strength and the water uptake behavior of such composites has been scarcely treated in the scientific literature. This paper uses fibers from old newspapers and obtain composite materials that are able to compete with commodity materials. The preparation of the composites avoids the use of reagents or deinking processes, which is aligned with the search for more environmentally friendly materials and processes.

## 2. Materials and Methods

### 2.1. Materials

The polymeric matrix was a polypropylene (PP) by Repsol YPF (Tarragona, Spain), with the commercial name ISPLEN^®^ PP09GM2. This is a homopolymer PP with a high melt flow index. This material is indicated for mold injection and allows being reinforced with high percentages of fiber. Its density is 0.906 g/cm^3^, and its melt flow rate (MFR) is 35 g/10 min (230 °C, 2.16 kg).

The coupling agent was PP maleated with maleic anhydride (MAPP), which was produced by Eastman Chemical Products (Middelburg, The Netherlands), under the commercial name EPOLENE^®^ G3015. This coupling agent has an acid number of 15 mg KOH/g and a molecular weight of 47,000 g/mol.

The reinforcement was obtained from the disintegration of old newspapers. Rotimpres (Girona, Spain) kindly provided the old newspapers, containing 85% of hardwood thermomechanical pulp and 15% of calcium carbonate as filler.

Clariant supplied the diethyleneglycol dimethyl ether (diglyme) used as the dispersing agent.

### 2.2. Old Newspaper Disintegration and Fiber Individualization

The old newspaper (ONPF) fibers were obtained by the disintegration of the newspapers in a pilot-scale pulper. Metrotec (Lezo, Spain) produced this equipment, which has the commercial reference Pucel Cell. The pulper has a volume of 20L and it is equipped with a helical rotor.

**Figure 1:**
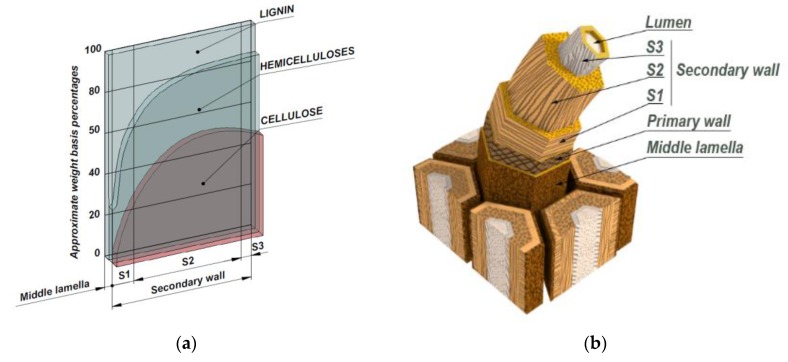
Chemical composition and structure of a typical lignocellulosic fiber: (**a**) Evolution of the chemical composition against depth; (**b**) Standard structure of a wood fiber.

The old newspapers were cut to 10 by 10 cm pieces and were put inside the pulper. The pulper’s tub was filled with water with 1% of NaOH. When the pieces were totally soaked, the rotor started and remained working for 20 min. The machine was operated at 20 rev/s rotor speed, a temperature of 50 °C, and 10% consistency. Afterwards, the obtained pulp was filtered and dried in an oven at 80 °C. In order to avoid the creation of hydrogen bonds between the hydroxyl groups of the cellulose and help the dispersion of the ONPF [40,41,42,43], the pulp was immersed in a water dyglime (1:3) solution. Figure 1a shows the standard chemical composition of a lignocellulosic fiber.

**Figure 2:**
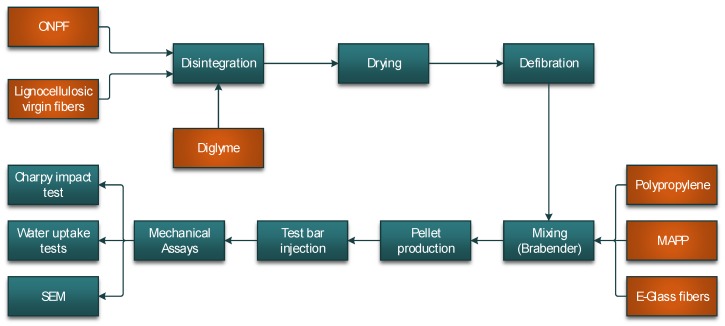
Flowchart of the experimental procedure.

Figure 1a shows how the chemical composition of a mechanical fiber, with a surface in the middle lamella, is mainly made of lignin, with lower percentages of cellulose and hemicelluloses. In the case of a thermomechanical fiber, some of the middle lamella is lost and the fiber surface is inside the S1 zone, increasing the cellulose and hemicellulose contents that allow the existence of hydroxyl groups. Figure 1b shows a typical structure of a lignocellulosic material. The bonds between the fibers must be broken in order to preview the formation of fiber bundles. Figure 2 shows a comprehensive flowchart of the experimental phases.

### 2.3. Composite Materials’ Preparation and Specimen Obtention

ONPF and GF-reinforced PP composites were prepared in a Brabender^®^ Plastograf internal mixing machine (Duisburg, Germany). The ONPF-based materials added 20 to 50 wt % of reinforcement, and the GF-based composites added 10 to 40 wt %. Two batches of ONPF-reinforced composites were prepared, one adding 6 wt % of MAPP and the other without any coupling agent. In the case of the ONPF composites, the mixer was operated at 80 rpm and 180 °C. The mixing lasted 10 min. The coupled GF-reinforced composites were mixed at 20 rpm due to the fragility of such reinforcements. All the resulting melts were pelletized in a knife mill. Prior to its mold injection, the pellets were dried during 1 h in a stove at 80 °C.

Standard rectangular specimens measuring 62 × 13 mm, with a thickness of 3.2 mm, in agreement with ASTM D638 [44] were mold injected. The injection molding machine was a Meteor 40 by Mateu & Solé (Barcelona, Spain). The machine has three heating areas, which were operated at 175, 175, and 190 °C, of which the last was the injection nozzle. The first and second pressures were 120 and 37.5 kgf/cm^2^, respectively. At least seven valid specimens for any of the composite formulations were mold injected. Five were devoted to the impact test and two were devoted to water uptake analysis.

### 2.4. Specimens Testing

Impact strength was carried out in agreement with the ISO 179-1:2010 standard [45]. Un-notched specimens were placed in a Charpy test equipment Instron Ceast 5.5 Resil by Ceast S.p.a. (Pianezza, Italy). The equipment was used to measure the energy absorbed during the impact.

Micrographs of the fractured surface of the specimens were obtained in order to observe possible fracture propagation clues. A DSM 960A Scanning Electron Microscope (SEM) by Zeiss (Madrid, Spain) was used for this purpose.

Before the water uptake measurements, the specimens were dried during 2 h in an oven at 150 °C. The moisture absorption of the samples was measured under two boundary conditions. One set was stored in a climatic chamber at 23 °C and 50% relative moisture atmosphere. This experiment was carried out in agreement with the ISO 62:2008 standard [46]. The other set was totally immersed in distilled water at 23 °C. The weight of the specimens was measured at regular intervals until saturation.

## 3. Results and Discussion

### 3.1. Impact Strength

The impact strength was obtained with a Charpy hammer and using un-notched specimens (ISO 179-1:2010) [45]. When the specimen is impacted with enough energy, a fracture is initiated, and then this fracture propagates. Thus, two phenomena are observed: on the one hand, the fracture creation, and on the other hand, the fracture propagation. The total energy needed to break the specimen, in the case of short fiber-reinforced composites, can be evaluated with the following equation [24]:

(1) $$w\approx w_{i}+w_{f}+w_{m}+\sum w_{fm}$$

where *w* is the total energy needed to fracture the specimen. The energies devoted to creating the initial fracture, breaking the fibers, and the matrix are represented by *w_i_*, *w_f_*, and *w_m_*, respectively. The energy needed to create the initial fracture can be linked to the plastic deformations preceding such fracture. This energy is affected by the properties of the phases and its relative contents, the morphology of the reinforcements, and the strength of the interface [12]. In the case of the fibers, the number of fractured fibers and their intrinsic strength affects the energy. The energy needed to fracture the matrix depends on its strength and the total surface of the propagation area. The rest of the energy ($\sum w_{fm}$) is related to the interactions between the fibers and the matrix (sliding, debonding, fiber pullout, etc.). These interactions can be very complex and are affected by the properties of the phases and the strength of the interface.

**Figure 3:**
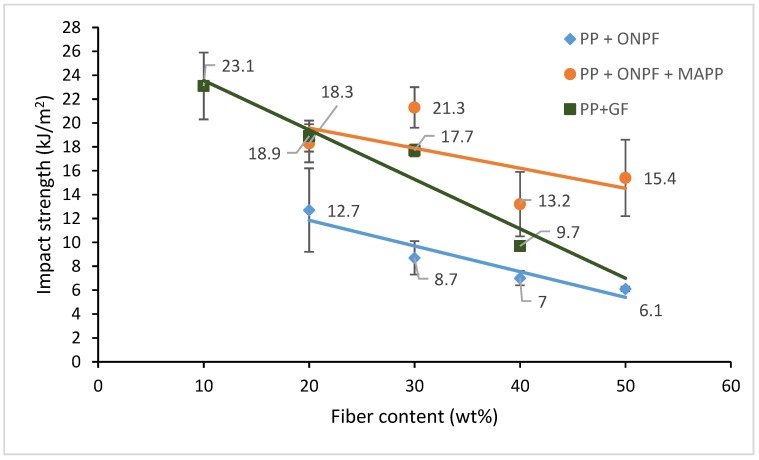
Charpy impact strength evolution regarding fiber content for uncoupled and coupled polypropylene (PP) + old newspaper (ONPF) and coupled PP + GF composites.

Figure 3 shows the evolution of the impact strength of the uncoupled and coupled ONPF-based composites against reinforcement content.

The test equipment was unable to break the neat PP specimen, showing its high toughness. In the literature, the impact strength of a PP is found to be in the vicinity of 85 kJ/m^2^ [47]. Nonetheless, as soon as a percentage of reinforcement was added to the composite, the impact strength decreased severely. Further amounts of reinforcement progressively decreased the impact strength of the composites. This decrease was less pronounced compared to the matrix. This can be due to the stress concentrations at the end of the fibers, which decrease the energy needed to create a fracture [15]. Therefore, the value of *w_i_* in Equation (1) is expected to decrease with the amount of reinforcement.

The impact strengths of the coupled composites were noticeably higher than the uncoupled ones. In the case of the coupled composites, there is an initial increase of the impact strength when the percentage of reinforcement was increased from 20 to 30 wt %. The literature shows similar behaviors for natural fiber-reinforced composites, with impact strengths increasing up to 30 wt % reinforcement contents. In a recent review of such cases, the authors blame the decreases on a possible decrease of the strength of the interface, fiber agglomerations that lead to stress concentrations, and to a reduction of the energy absorption due to fiber–fiber contact. Similar behaviors have reported in the literature [7,48].

The differences between the impact strengths of uncoupled and coupled composites can be related with a change in the mechanism of fracture creation and propagation. Initial fractures will be developed in the feeblest phase, which latter propagate accordingly to the mechanisms described in the literature [49].

**Figure 4:**
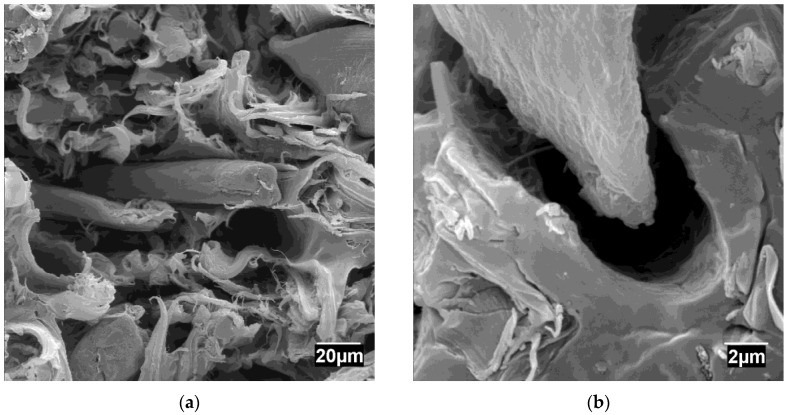
SEM micrographs of uncoupled PP composites reinforced with 40 wt % at different resolutions: (**a**) Detail of the dispersion of the ONPF fibers in the matrix and some possible pullout fibers; (**b**) Detail of a void in the fiber matrix interface.

Figure 4 shows micrographs of the fractured surface of an uncoupled composite.

Figure 4a shows reinforcing fibers dispersed in the matrix. Figure 4a shows the presence of individualized fibers. The figure does not evidence any fiber/fiber contacts backing up the hypothesis of a good fiber dispersion. In the micrograph are visible fibers and voids. These voids can indicate a pulled-out fiber. Figure 4b shows a detail of the interface between the matrix and the fibers. A void all around the fiber was observed showing the expected low adhesion between hydrophilic lignocellulosic fibers and hydrophobic polyolefin matrices. The literature proposes three main crack propagation cases: through the matrix, the dispersed phase, or the interface [49]. The fibers have detached from the matrix when the stress was applied, and under these conditions, the fracture propagates through the interface [49]. Due to the low strength of the interfaces, the fibers are pulled out, needing low amounts of energy. The figure shows fibers without rugose surfaces, which decreases the mechanic anchoring of such fibers to the matrix.

Compared to glass fiber-reinforced PP, GF shows a regular shape, which is typical of manmade fibers. These fibers also show higher lengths when detached from the matrix, which is mainly due to its higher tensile strength [50].

**Figure 5:**
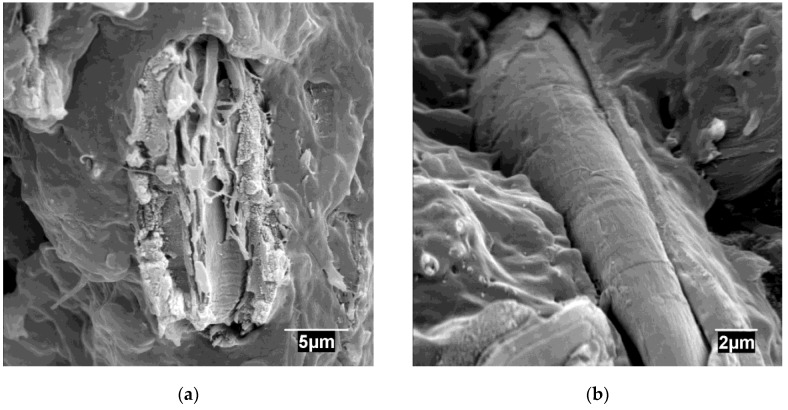
SEM micrographs of coupled PP composites reinforced with 40 wt % at different resolutions: (**a**) Detail of a brook fiber and the absence of voids in the interface; (**b**) Detail of the interface.

Micrographs of the fractures surfaces of coupled composites (Figure 5) display noticeable differences from those of uncoupled materials (Figure 4).

The presence of MAPP increased the impact strength of the composites. This is in line with the literature, where this increase has been related to the strength of the interface of coupled composites [31]. There are no voids all around the fiber, and the matrix totally wets the fiber (Figure 5a). Thus, in addition to chemical bonding, a higher mechanical anchoring is expected [18,19,51]. The figure shows a broken fiber changing the propagation of the fracture mechanism [49]. The interface is not strong enough to grant a crack propagation through the matrix. Figure 5a shows a fiber broken in the same plane as the matrix; thus, the fibers have broken before detaching from the matrix [49]. Then, supercritical fibers will break and subcritical fibers will pull out. Due to the higher adhesion, the pullout of the fibers needs a continuous application of energy. Figure 5a shows a fiber oriented almost parallel to the fractured surface. The fracture has teared out the matrix, leaving the fiber embodied in the matrix. In this case, the fracture has propagated on the interface, as it is less strong than the fiber. Similar propagation mechanisms have been reported for other natural fiber-reinforced composites [23,25,30]. In the case of GF-reinforced composites, the addition of coupling agents, at proper percentages, also increases the impact strength and the energy absorption [50].

Regarding Equation (1), the main differences between coupled and uncoupled composites will be related to the contribution of the fibers and the interactions between the fibers and the matrix. On the one hand, the contribution of the fibers (*w_f_*) in the uncoupled composites is expected to be very low, as the fibers have apparently slipped out the matrix, and few fibers appear to have broken. Coupled composites show clearly broken fibers; thus, the fibers have contributed to the impact strength of the material. The interactions between the fibers and the matrix are expected to be low in the case of uncoupled composites, which is mainly due to the low strength of its interface. In this case, the interface adds a discontinuous area that decreases the strength of the material. Coupled materials do not add such discontinuities, and the fracture propagates from the matrix to the fiber through the interface. Energy must be devoted to pulling out the reinforcement. Moreover, coupled composites show higher scatter than uncoupled composites in their impact strength. This can be due to the high scatter of the intrinsic properties of natural fibers [48]. The properties of a composite are the results of the contributions of the phases. Therefore, if one of the constituents of a composite shows a noticeable scatter on a property, the scatter of such a property for the composite will reflect this scatter. Equation (1) models the impact strength of the composite as a sum of contributions. In the case of uncoupled composites, the contribution of the fibers is scarce, and the scatter of its intrinsic properties has little impact on the property of the composite. On the other hand, the scatter of natural fibers had a high impact on the scatter of the strength impact of coupled composites.

In order to compare ONPF-reinforced composites with commercial commodities, Figure 3 displays the impact strengths of ONPF composites with GF-reinforced PP materials.

In the case of uncoupled composites, at low reinforcement contents, GF-reinforced composites are noticeably stronger than ONPF composites under impact conditions. The impact strength of a 20 wt % reinforced composite is 49% higher than its ONPF-reinforced counterpart. Nonetheless, the differences between the impact strengths of both composites decreased with the amount of reinforcement. The regression line of the GF-reinforced composites showed a higher slope than the ONPF materials. In this sense, theoretically, composites with 60 wt % reinforcement GF and ONPF reinforcement contents will show similar impact strengths. This is only theoretical because, on the one hand, such amounts of reinforcement will increase notably the MFI of the composites, hindering its mold injection. On the other hand, a correct dispersion of such amount of reinforcement is difficult. It must be noted that commercial GF-reinforced composites have reinforcement contents that are usually in the range from 10 to 30 wt %. Thus, the relevant impact strengths are those of such materials. Uncoupled ONPF composites were unable to reach these specifications.

Coupled ONPF composites showed competitive impact strengths. The ONPF composites showed higher impact strengths than that of the 40 wt % GF-reinforced composite (Figure 3). This impact strength also compares well with a 20 wt % GF material. Thus, ONPF-reinforced composites and GF-reinforced materials can return similar impact strengths. The composite with 50 wt % ONPF content shows an impact strength that is only 15% inferior to a 30 wt % GF-reinforced material, which is a commercial referent. This material avoids using a mineral reinforcement that needs high amounts of energy to be manufactured, as opposed to a recycled fiber. Moreover, old newspapers are widely available, avoiding further long-distance transports of materials. In addition, adding more natural fiber phase decreases the amount of oil-based matrix. Thus, these materials, showing similar impact strengths, are, a priory, more environmentally friendly.

### 3.2. Water Uptake Behavior of ONPF-Reinforced Composites

**Figure 6:**
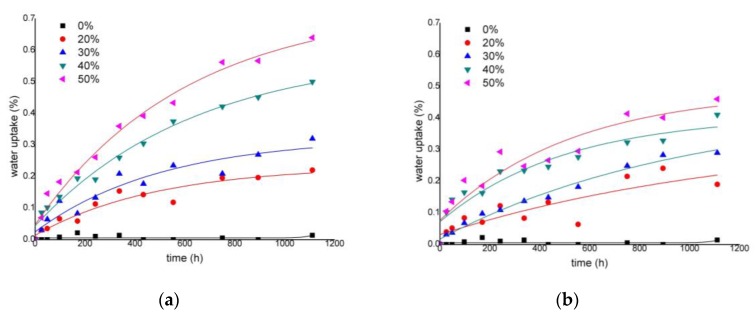
Water uptake profiles of composites reinforced with different percentages of ONPF at 23 °C and 50% RH: (**a**) Uncoupled ONPF composites; (**b**) Coupled ONPF composites.

The water uptake of the composite materials was explored under two different conditions, on the one hand at 23 °C and 50% relative moisture atmosphere, and on the other hand under total immersion in distilled water at 23 °C. Two different sets of specimens were tested: on the one hand, a mold-injected specimen as extracted from the mold, and on the other hand, a specimen broken into five pieces. The broken pieces showed unexpected water uptake behaviors that did not agree with the literature. The authors blame this deviation on the differences between the mold surfaces and the broken ones. The presence of a more rough and porous surface, and some fiber ends or detached fibers created additional water absorption canals that changed the expected results. Therefore, only the results obtained with the unbroken specimens are shown. Moisture sorption was obtained by measuring the mass of the specimens against time. Figure 6 shows the curves with the percentage of absorbed water against time for the uncoupled and coupled ONPF-reinforced materials.

Uncoupled materials showed a noticeable increase of its water uptake when the percentage of ONPF increased. After 46 days of exposure, the materials reinforced with 20, 30, 40, and 50 wt % of ONPF showed 0.22%, 0.32%, 0.50%, and 0.64% water uptake percentages, respectively (Table 1). Figure 6 show the negligible water uptake of the matrix. Thus, hydrophilic fibers are responsible for the water uptake of the composites [52,53].

The presence of a coupling agent in the formulation of the composites affected the percentages of water uptake. After 46 days, the coupled composites showed 0.19%, 0.29%, 0.41% and 0.46% water uptake percentages, respectively. With respect to uncoupled materials, the percentage decreases were 16%, 9%, 17%, and 28% at 20 to 50 wt % ONPF contents, respectively. This can be due to the increased adhesion between the phases and the absence of voids in the interfacial area, hindering the diffusion and accumulation of water in such voids (Figure 4b and Figure 5a). On the other hand, the hydrophilicity of the fibers is reduced due to the presence of MAPP, as some fiber surface hydroxyl groups create ester bonds with the coupling agent [54]. Other studies found similar effects regarding the presence of coupling agents, where the initial water uptake rates are inferior in the case of coupled composites, but the presence of such coupling agents has little effect on the maximum moisture content [55].

**Figure 7:**
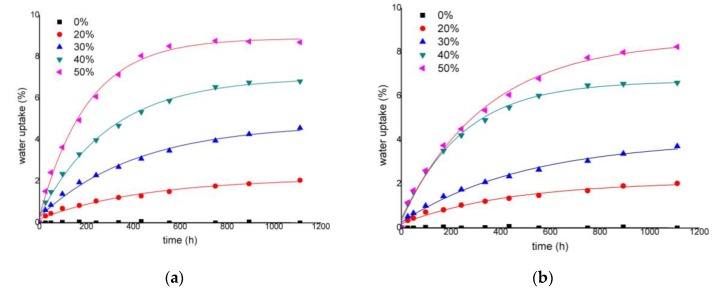
Water uptake profiles of composites reinforced with different percentages of ONPF under water immersion conditions: (**a**) Uncoupled ONPF composites; (**b**) Coupled ONPF composites.

A second set of specimens of the same materials were totally immersed in water at 23 °C. Figure 7 shows its water uptake behavior.

The behavior was similar, and the water uptake percentage increased with the amount of reinforcement, but the percentages of water uptake increased under immersion conditions. In this sense, after 46 days of immersion, uncoupled composites with 20 to 50 wt % ONPF contents showed 2.1%, 4.6%, 6.8%, and 8.7% water uptakes, respectively (Figure 7).

Coupled materials have a decreased water uptake (Figure 7b). The reasons are the same as those already discussed for materials exposed to humid conditions. In this case, coupled materials decreased their water uptake by 1%, 19%, 3% and 5% with respect to coupled ones at 20 to 50 wt % ONPF contents.

The main mechanism governing water uptake is water molecules’ diffusion into composite micro gaps. In addition, there is capillarity phenomena that allow the access of the water to the voids in the interface and water transport among the micro-breakages created in the matrix during mold injection [9,56]. Although all these mechanisms are present at the same time, the effect can be modeled taking only the diffusion in account.

The diffusion behavior of a material comprehends four cases: Fickian diffusion, Case II, Super Case II, and Non-Fickian diffusion. These behaviors can be identified from the shape of the sorption curves (Figure 6 and Figure 7). Equation (2) shows its mathematical shape. Equation (3) shows a linearized version.

(2) $$\frac{M_{t}}{M_{\infty }}=kt^{n}$$

(3) $$\log\left(\frac{M_{t}}{M_{\infty }}\right)=\log k+n\log t$$

In the equations, *M_t_* is the water content at a defined time *t*. *M*_∞_ is the equilibrium state, and *k* and *n* are kinetic constants. *M_t_* can be obtained from:

(4) $$M_{t}=\frac{m_{t}-m_{0}}{m_{0}}\cdot 100$$

where *m*_0_ and *m_t_* are the mass of the specimen at the beginning and before t hours of experiment, respectively.

The parameter *n* defines the sorption case. This constant takes values are near 0.5 for Fickian diffusion, near 1 for Case II, higher than 1 for Super Case II, and values between 0.5 and 1 for a Non-Fickian behavior.

Table 1 shows the computed values for the *n* and *k* constants, which were obtained from the curves shown in Figure 6 and Figure 7.

The *n* value was obtained from the slope of the linear shape of the curves (Equation (3)). All the values were close to 0.5, suggesting a Fickian behavior. This is in line with other natural fiber-reinforced PP composites where Fickian behavior was identified as the sorption case [23,34,57]. This allows using Equation (6) to obtain a diffusion coefficient (*D*). This equation can be used for a small time-lapse (*M_t_*/*M*_∞_ < 0.5).

(5) $$\frac{M_{t}}{M_{\infty }}=\frac{4}{L}{\left(\frac{D}{\pi }\right)}^{0.5}t^{0.5}$$

This diffusion coefficient defines the capacity of the solvent to penetrate a material. In the equation, *L* is the sample thickness. Equation (6) refers to an infinite solid. Therefore, Equation (6) was considered more suitable. The equation has in account diffusion phenomena that occur in the specimen edges.

(6) $$D_{c}=D{\left(1+\frac{L}{H}+\frac{L}{W}\right)}^{-2}$$

*D_c_* is a compensated diffusion coefficient; *H* is the length of the specimen; and *W* is its width. Table 1 shows the obtained values for the diffusion and the compensated diffusion coefficients. The diffusion coefficient increased with increasing amounts of reinforcement. In other words, the presence of a lignocellulosic reinforcement increased the speed of water penetration in the material. The main reason is due to the hydrophilic nature of ONPF [53], opposed to the hydrophobic nature of the matrix. It is worth noting how immersed materials showed diffusion coefficients that are two times higher than the materials exposed to a humid atmosphere. The use of coupling agents delayed the permeation of the water among the interface due to the absence of voids in such an interface. The compensated diffusion coefficients of the coupled materials under humid conditions were 39%, 22%, 13%, and 27% lower than those of the uncoupled materials for ONPF contents ranging from 20 to 50 wt %. The literature shows similar results in the case of other natural fiber-reinforced composites in terms of diffusion behavior [22,24,34,35]. The literature shows the effect of treatments of the diffusion coefficient. Different treatments on sisal fibers reduced the diffusion coefficient and similar to the ONPF composites, the diffusion coefficient increased with the fiber content and decreased when fiber treatments were applied [34]. This was also noticed in other studies that studied the relation between water sorption behavior and fiber chemical composition [58].

## 4. Conclusions

Composite materials made of polypropylene reinforced with old newspaper recycled fibers were formulated, produced, and tested under a Charpy impact test and water uptake conditions. The effect of adding coupling agents on such material properties were also explored.

Adding ONPF decreased noticeably the impact strength of the composites. The impact strength of the composites decreased when the percentage of ONPF was increased. The presence of coupling agents in the formulation of the composites increased the impact strength of the materials. This increase was related with the mechanism of fracture propagation. In the case of uncoupled composites, the contribution of the fibers and the interactions between the fibers and matrix were considered feeble due to the low strength of the interface. Thus, the fracture was hypothesized to propagate through the interface. In the case of coupled composites, the strength of the interface ensures fiber contributions and the presence of interactions between fibers and matrix in the shape of energy devoted to fiber pullout. The fracture propagation was through the matrix, with fiber breakage and pullout phenomena.

Coupled composites showed an impact strength that was in line with that of the glass fiber-reinforced PP materials. ONPF composites at 50 wt % contents showed impact strengths higher than a 40 wt % GF-reinforced composite and slightly lower than a 30 wt % GF material. This can be seen as an environmental advantage of ONPF-based materials, as they replace a mineral reinforcement with a natural fiber and decrease the percentage of the oil-based matrix.

The percentage of reinforcement had a noticeable effect on the water uptake of the materials. This water uptake increased with the amount of reinforcement. It was found that the percentage of ONPF did not affect the diffusion coefficients, showing the dependence of such coefficients on the chemical structure of the phases and its independence from the weight fractions of such phases. The use of coupling agents decreased the water uptake of the composites. Thus, coupled composites can show better behaviors under humid conditions than uncoupled ones.

The research shows the possibilities of composite materials reinforced with recycled fibers from waste old newspaper.

## Figures and Tables

**Table 1:** Parameters of Fick’s Law and moisture diffusion coefficient of PP composites reinforced with ONPF. MAPP: maleated with maleic anhydride.

Condition Parameters	MAPP (%)	Fiber Content (%)	*M*_∞_ (%)	*n*	*k* (10^−4^ s^−1/2^)	*D* (10^−13^ m^2^∙s^−1^)	*D_c_* (10^−13^ m^2^∙s^−1^)
23 °C, 50% HR	0	20	0.22	0.52	3.23	3.29	1.51
		30	0.32	0.57	1.60	3.41	1.56
		40	0.50	0.46	8.24	3.75	1.72
		50	0.64	0.53	2.96	4.59	2.10
	6	20	0.19	0.44	1.08	2.03	9.30
		30	0.29	0.55	1.83	2.65	1.21
		40	0.41	0.48	1.08	3.26	1.50
		50	0.46	0.47	1.04	3.63	1.66
23 °C, water immersion	0	20	2.05	0.50	5.61	4.83	2.21
		30	4.57	0.58	1.92	6.73	3.09
		40	6.82	0.52	4.60	9.19	4.21
		50	8.70	0.56	3.49	1.27	5.83
	6	20	2.03	0.47	8.25	4.72	2.17
		30	3.72	0.52	3.96	5.38	2.47
		40	6.61	0.57	2.56	7.61	3.49
		50	8.24	0.58	2.09	7.96	3.65
